# Characteristics of cadmium accumulation and tolerance in apple plants grown in different soils

**DOI:** 10.3389/fpls.2023.1188241

**Published:** 2023-06-02

**Authors:** Xiaolei Zhuang, Huixue Wan, Hongyu Wang, Sijun Qin, Jiali He, Deguo Lyu

**Affiliations:** ^1^ College of Horticulture, Shenyang Agricultural University, Shenyang, Liaoning, China; ^2^ Key Lab of Fruit Quality Development and Regulation of Liaoning Province, Shenyang Agricultural University, Shenyang, Liaoning, China

**Keywords:** apple, cadmium, soil physicochemical property, oxidative stress, gene expression

## Abstract

Cadmium (Cd) is a nonessential element and highly toxic to apple tree. However, Cd accumulation, translocation and tolerance in apple trees planted in different soils remain unknown. To investigate soil Cd bioavailability, plant Cd accumulation, physiological changes as well as gene expression patterns in apple trees grown in five different soils, ‘Hanfu’ apple seedlings were planted in orchard soils collected from Maliangou village (ML), Desheng village (DS), Xishan village (XS), Kaoshantun village (KS) and Qianertaizi village (QT), and subjected to 500 μM CdCl_2_ for 70 d. Results showed that soils of ML and XS had higher content of organic matter (OM), clay and silt, and cation exchange capacity (CEC) but lower sand content than the other soils, thereby reduced Cd bioavailability, which could be reflected by lower concentrations and proportions of acid-soluble Cd but higher concentrations and proportions of reducible and oxidizable Cd. The plants grown in soils of ML and XS had relatively lower Cd accumulation levels and bio-concentration factors than those grown in the other soils. Excess Cd reduced plant biomass, root architecture, and chlorophyll content in all plants but to relatively lesser degree in those grown in soils of ML and XS. The plants grown in soils of ML, XS and QT had comparatively lower reactive oxygen species (ROS) content, less membrane lipid peroxidation, and higher antioxidant content and enzyme activity than those grown in soils of DS and KS. Transcript levels of genes regulating Cd uptake, transport and detoxification such as *HA11*, *VHA4*, *ZIP6*, *IRT1*, *NAS1*, *MT2*, *MHX*, *MTP1*, *ABCC1*, *HMA4* and *PCR2* displayed significant differences in roots of plants grown in different soils. These results indicate that soil types affect Cd accumulation and tolerance in apple plants, and plants grown in soils with higher OM content, CEC, clay and silt content and lower sand content suffer less Cd toxicity.

## 1 Introduction

Extensive sewage irrigation and metal-based pesticide and chemical fertilizer application in recent years have contaminated fruit orchards with cadmium (Cd) to varying degrees (Li et al., 2006; Karimi Nezhad et al., 2011; Argüello et al., 2019). Cd is water-soluble and readily transported from the soil to roots (Luo et al., 2016; He et al., 2020). In plants, Cd induces reactive oxygen species (ROS) production, causes oxidative stress, destroys subcellular structure, reduces photosynthesis, inhibits growth, and may cause death (Liu and Kottke, 2004; Mobin and Khan, 2007; Podazza et al., 2012; Podazza et al., 2016). Moreover, Cd in agricultural products may be ingested by humans and cause various diseases such as hypertension, kidney dysfunction and lung cancers (Chen et al., 2015).

The Cd content in plant tissues depends largely upon the Cd bioavailability in the soil and the ability of the plants to absorb soil Cd. Therefore, it is important to understand the factors affecting Cd migration from the soil to roots. After Cd enters the soil, it undergoes physicochemical transformations such as dissolution, precipitation, complexation, and adsorption and assumes different forms (Qasim and Motelica-Heino, 2014). Cd bioavailability in plants is controlled by various soil physicochemical factors, such as pH value, organic matter (OM) content, soil texture, and cation exchange capacity (CEC) (Golia et al., 2008; Karimi Nezhad et al., 2011). This relationship has been established in some crops where soil Cd mobility and plant Cd uptake are decreased at high soil pH, OM content and CEC (Vega et al., 2010; Chen et al., 2015; Chen et al., 2016; Argüello et al., 2019). In addition, soil texture is also an important factor affecting heavy metal bioavailability. Finer soil particles have relatively larger surface areas, higher secondary mineral and OM content (Hardy and Cornu, 2006; Yu et al., 2017), and greater heavy metal adsorption capacity (Luo et al., 2011). Soils of fruit orchards differ greatly in terms of these key properties in different planting regions. However, little is known about Cd accumulation and toxicity in apple trees planted in different soils.

Excess Cd induces the production of ROS including superoxide anion ( ${\text{O}}_{2}^{\;\cdot -}$ ) and hydrogen peroxide (H_2_O_2_) which cause cell membrane lipid peroxidation, nucleic acid and protein denaturation, abnormal cell metabolism, and even cell death (PÉRez-Chaca et al., 2014; Ahammed et al., 2021). Plants activate their antioxidant defense systems including antioxidant enzymes and non-enzymatic antioxidant metabolites to mitigate oxidative damage induced by Cd (Anjum et al., 2015; Zhou et al., 2017). The antioxidant capacity of soybean under heavy metal stress displayed significant differences when planted in different soils (Melo et al., 2011). In addition, application of organic amendments affected the antioxidant defense system and alleviated Cd toxicity in mung bean (Ramzani et al., 2017), possibly because they altered the pH, CEC, OM, and other physicochemical properties of the soil (Fellet et al., 2014; Cui et al., 2016). To our knowledge, however, little is known about how Cd stress affects the antioxidant defense system of apple trees grown in different soils.

Plant Cd absorption, transport, and detoxification are key processes controlled by several genes (He et al., 2020). Plasma membrane (PM) H^+^-ATPase pumps protons extracellularly at the expense of ATP and creates an electrochemical gradient for transmembrane ion transport (Zhang et al., 2017). Upregulation of the gene encoding PM H^+^-ATPase enhanced Cd^2+^ absorption in plants (He et al., 2015). ZRT-IRT-like protein 6.2 (ZIP6.2) and iron transporter 1 (IRT1) are plant cells membrane localized protein and regulate extracellular Cd^2+^ uptake (Migeon et al., 2010; Zhu et al., 2012). Upon entry into root cells, Cd may complex with Cd effective chelators including nicotianamine (NA; encoded by nicotianamine synthase 1 (NAS1)) and metallothionein (He et al., 2020). Metal tolerance protein 1 (MTP1), magnesium proton exchangers (MHX), and ATP-binding cassette transporter C1 (ABCC1) are localized to the tonoplasts. MTP1 and MHX transport Cd^2+^ and ABCC1 transports PC-Cd complexes into the vacuoles (Berezin et al., 2008; Gaillard et al., 2008; Migeon et al., 2010). Plant Cd resistance protein 2 (PCR2) and HM ATPase4 (HMA4) are localized to the PMs, pump Cd into the apoplast, and play critical roles in Cd translocation from the roots to the aerial parts (Hanikenne et al., 2008; Song et al., 2010). Up to now, little is known about the gene expression patterns related to Cd absorption, transport and tolerance in apple plants grown in different soils.

‘Hanfu’ apple (*Malus domestica* Borkh.) is a high quality variety with cold resistance and high yield, and has become the main variety grown in Liaoning Province. In this study, according to the different characteristics of the apple orchard soil in the Liaoning Province, orchards soils in five regions were collected. To explore the differences of Cd absorption, accumulation and tolerance of apple seedlings grown in different soils, ‘Hanfu’ apple grafted onto *M. baccata* Borkh. with relative higher Cd tolerance (Zhou et al., 2017) were planted in five soils with different physicochemical properties and subjected to 500 μM Cd for 70 d. We hypothesized that (i) there would be variations in Cd accumulation and tolerance in apple plants grown in different soils, and (ii) these variations are associated with physiological acclimation and transcriptional regulation. To examine these hypotheses, we compared and analyzed various soil Cd forms, growth characteristics of plants, Cd concentrations, ROS, antioxidants, and the transcription level of key genes related to Cd uptake, transport, and detoxification. The obtained results are of great significance for mitigating the risk of Cd phytotoxicity in apple as well as other fruit trees through the application of amendments to modify soil properties.

## 2 Materials and methods

### 2.1 Soil sources and physicochemical properties

Soils were sampled at 0–20 cm depth from ‘Hanfu’ apple (*Malus domestica* Borkh.) orchards in Maliangou village in Liaoyang city (ML), Desheng village in Panjin city (DS), Xishan village in Chaoyang city (XS), Kaoshantun village in Xinmin city (KS), and Qianertaizi village in Xinmin city (QT) in Liaoning Province, China. Visible residues were removed from the samples and they were then passed through a 2-mm mesh sieve and air-dried for the subsequent experiment.

The pH value was determined for each 1:2.5 (soil/water (w/v)) suspension by pH meter (PHS-3C; Shanghai Leici Equipment Factory, China). OM was analyzed according to Sparks et al. (1996). CEC was measured as described previously (Gaskin et al., 2008). The content of clay, silt, and sand were determined using a laser particle size analyzer (MS3000; Malvern PANanalytical, Malvern, UK). The initial soil Cd concentration was measured by the procedure described by Wu et al. (2014).

### 2.2 Plant material and Cd exposure

Full buds on branches of ‘Hanfu’ apple were grafted on annual seedlings of *M. baccata* Borkh., and planted in a plastic pots (one plant pot^-1^) containing 2.5 kg soil in a greenhouse with natural photoperiod (day/night temperature, 25/17°C; relative air humidity, 50%–60%), and watered every dusk. When the plants grew to about 20 cm, 120 plants with similar heights were divided equally to ten groups, and transplanted them in different soils collected from five regions. After two weeks, 100 mL of 500 μM CdCl_2_ or distilled water was irrigated to the plants of each group in every day, and the final soil Cd content was 156.8 mg kg^-1^. A plastic tray was placed under each pot to collect the soil leachate which was poured back into the pot to prevent Cd leaching. The soil was regularly irrigated with distilled water, and the soil moisture of each pot was maintained at approximately 70% of field water capacity. The concentration of Cd treatment was used on the basis of previous study (Abdel Latef, 2013). Three replicates for each treatment were conducted, and each replicate contained 4 plants. Plants were harvested after Cd treatment for 70 d.

### 2.3 Soil collection and plant harvest

The rhizosphere soil of each treatment was collected by shaking off the roots in the air, and then air-dried and then passed through a 2-mm mesh sieve. The dust and soil on the surface of plants were washed with deionized water, and then the roots of each plant were rinsed in 50 mM CaCl_2_ solution for 5 minutes to remove Cd from the root surface (He et al., 2013b). Subsequently, the roots were carefully washed with deionized water (He et al., 2013b). After separating into the roots, stems and leaves and recording their fresh weight, different tissues were immediately frozen in liquid nitrogen. The frozen samples were then ground to fine powder using a ball mill (MM400; Retsch, Haan, Germany) and subsequently stored at -80°C. The fresh material of each tissue per plant was dried at 60°C for 72 h to analyze the fresh-to-dry mass ratio.

### 2.4 Determination of Cd forms in soil

The content of Cd chemical forms including acid-soluble Cd, reducible Cd, oxidizable Cd and residual Cd in soil samples was measure by European Community Bureau of Reference (BCR) sequential extraction method (Sungur et al., 2014b). After digestion of residues with a mixture of acids [HNO_3_/H_2_O_2_/HF (3/2/1 (v/v/v)] using a microwave digestion system (MARS 6 CLASSIC; CEM Corporation, Matthews, NC, USA) as described by Wu et al. (2014), the Cd levels in the extract and digest were measured using a flame atomic absorbance spectrometry (FAAS; Hitachi 180-80; Hitachi Ltd., Tokyo, Japan).

### 2.5 Analysis of root characteristics and photosynthetic pigments

The WinRHIZO Root Analyzer System (WinRHIZO 2012b; Regent Instruments Canada Inc., Montreal, Canada) was used to measure the total root length, total root surface area and total root volume based on the method of Ma et al. (2014). Chlorophyll and carotenoids contents in the leaves of plants were determined using a spectrophotometer (UV-3802; Unico Instruments Co. Ltd, Shanghai, China) according to the method of He et al. (2011).

### 2.6 Analysis of Cd concentration, total Cd and bio-concentration factor (BCF)

Frozen samples (ca. 100 mg) of each tissue were digested at 170°C in a mixture containing HNO_3_/HClO_4_ (7/1 (v/v)) as previously described (Zhou et al., 2016). The content of Cd in each tissue was determined by flame atomic absorption spectrometry (Hitachi 180-80; Hitachi Ltd, Tokyo, Japan). The total Cd amounts of each tissue were calculated based on the concentration of Cd in each tissue multiplied by the dry mass of the corresponding tissue. BCF was calculated using Cd concentration (μg g^-1^ DW) in root or aerial parts tissues divided by the Cd concentration (μg g^-1^ DW) in the soil (He et al., 2015).

### 2.7 Measurement of O_2_ ^·−^, H_2_O_2_ and MDA

The ${\text{O}}_{2}^{\;\cdot -}$ and H_2_O_2_ concentrations of each tissue were determined spectrophotometrically at 530 and 410 nm, respectively, as recommended by He et al. (2011). According to Lei et al. (2007), the MDA concentrations in each tissue of plants were analyzed at 450, 532 and 600 nm with a spectrophotometer.

### 2.8 Analysis of non-enzymatic and enzymatic antioxidants

The contents of non-enzymatic metabolites including free proline, ascorbate (ASC), T-SH and soluble phenolics in each tissue of plants were measured by the procedure described by He et al. (2013a).

The soluble protein contents in each sample were measured by the procedure described by Luo et al. (2008). The activities of SOD, CAT, POD and ascorbate peroxidase (APX) were measured as previously described by He et al. (2011), and the activities of glutathione reductase (GR) were determined as described by Wang et al. (2013).

### 2.9 Analysis of gene transcription levels

According to Zhou et al. (2016), the transcript level of genes was analyzed. Root total RNA was extracted and purified based on the manufacturer’s instructions by a RNA extraction kit (R6827, Omega Bio-Tek, Norcross, GA, USA). Then a spectrophotometer (NanoDrop 2000; Thermo Fisher Scientific, Waltham, MA, USA) and agarose gel electrophoresis was used to determine the concentration and quality of total RNA, respectively. Subsequently, 1 μg of total RNA was used to synthesize first-strand cDNA using a PrimeScript RT Reagent Kit with gDNA Eraser (DRR037A, Takara, Dalian, China) following the manufacturer’s protocol. Quantitative PCR for each gene was run with 10 μL 2× SYBR Green Premix Ex Taq II (DRR820A, Takara), 0.5 μL cDNA and 0.2 μM each gene-specific primer (**Table S1**). *β-Actin* was used as a reference gene (**Table S1**). 2^-ΔΔCT^ method was used to calculate the relative mRNA expression. For each gene in the roots, expression level was set to 1 in ‘Hanfu’ apple plants grown in soil of ML subjected to 0 μM CdCl_2_. Corresponding fold changes in other treatments were then calculated accordingly. Gene expression heatmap was generated based on log_2_ average expression fold values.

### 2.10 Statistical analysis

All statistical analyses were processed using Statgraphics (STN, St Louis, MO, USA) after confirming their normality. For all physiological parameters, two-way ANOVA was employed using Cd treatment (Cd) and soil (S) as factors. To reduce the error of type I, Tukey-HSD method was used to correct the *P*-value obtained from multiple comparisons. When the *P*-value was < 0.05, differences between treatment means were considered significant. Pearson’s correlation analysis was used to determine the relationships between soil Cd forms and soil properties as well as Cd content in plant tissues. For the principal component analysis (PCA), the data of plant growth related parameters, photosynthetic pigments, Cd concentrations, ROS and antioxidant levels in the roots, stems, and leaves were standardized and subsequently computed by the command prcomp () in R (http://www.r-project.org/), as suggested by He et al. (2015).

## 3 Results

### 3.1 Soil physicochemical properties

There were significant differences among the five soil types in terms of their physicochemical properties and initial Cd content (**Table 1**). The pH value of soil of KS was the lowest while those of soils of DS and QT were considerably higher than those of all others. Soils of DS, KS, and QT had relatively lower OM content, CEC, clay and silt content and relatively higher sand content than soils of ML and XS. Initial Cd content was higher in soil of XS than that in all others (**Table 1**).

**Table 1:** Physicochemical properties of 0–20 cm soil samples collected from five apple orchards.

Soils	pH	OM (%)	CEC (cmol kg^-1^)	Clay (%)	Silt (%)	Sand (%)	Cd (μg g^-1^)
ML	5.82 ± 0.02 b	1.51 ± 0.09 a	21.81± 0.63 a	4.05 ± 0.53 b	56.05 ± 1.52 a	39.91 ± 1.10 c	0.20 ± 0.01 ab
DS	6.62 ± 0.03 a	1.08 ± 0.05 c	5.41 ± 0.11 c	0.95 ± 0.11 d	13.11 ± 0.69 d	85.94 ± 0.81 a	0.14 ± 0.01 b
XS	6.06 ± 0.17 b	1.61 ± 0.08 a	21.29± 0.92 a	22.96 ± 0.57 a	34.89 ± 0.65 b	42.15 ± 0.12 c	0.27 ± 0.01 a
KS	4.88 ± 0.10 c	1.36 ± 0.05 b	7.05 ± 0.43 bc	1.52 ± 0.13 c	17.62 ± 1.08 c	80.86 ± 1.22 b	0.20 ± 0.04 ab
QT	6.76 ± 0.05 a	1.32 ± 0.03 b	9.96 ± 0.35 b	0.95 ± 0.02 d	11.87 ± 0.74 d	87.19 ± 0.75 a	0.16 ± 0.02 b

Different letters beside values in same column indicate significant differences between soils. ML, Maliangou village in Liaoyang city. DS, Desheng village in Panjin city; XS, Xishan village in Chaoyang city; KS, Kaoshantun village in Xinmin city; QT, Qianertaizi village in Xinmin city; OM, organic matter; CEC, cation exchange capacity. Data are means ± SE (n = 3).

### 3.2 Cd content and proportion in various soils

**Figure 1:**
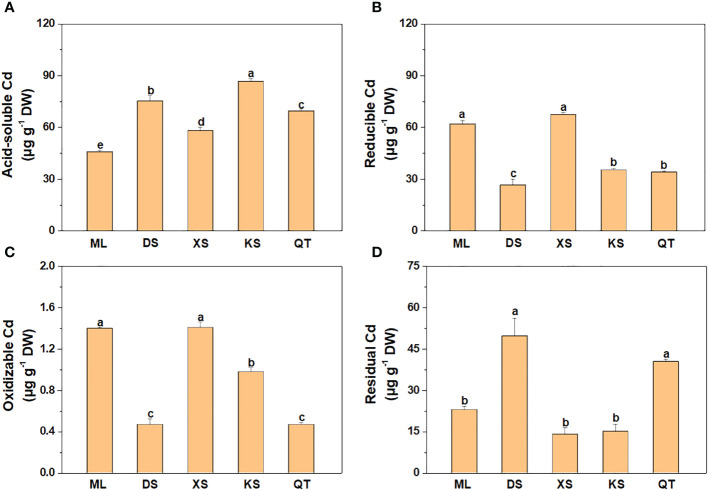
Cd concentrations of acid-soluble **(A)**, reducible **(B)**, oxidizable **(C)**, and residual **(D)** forms in soils sampled from five apple orchards subjected to 500 μM CdCl_2_ for 70 d. Data are means ± SE (n = 3). Different letters on bars indicate significant differences between soils. ML, Maliangou village in Liaoyang city; DS, Desheng village in Panjin city; XS, Xishan village in Chaoyang city; KS, Kaoshantun village in Xinmin city; QT, Qianertaizi village in Xinmin city.

The acid-soluble Cd content was lower in soils of ML and XS than soils of DS and KS (**Figure 1A**). However, the reducible and oxidizable Cd contents in soils of ML and XS were dramatically higher than those in the other soils (**Figures 1B, C**). The residual Cd content was remarkably higher in soils of DS and QT than the other soils (**Figure 1D**).

**Figure 2:**
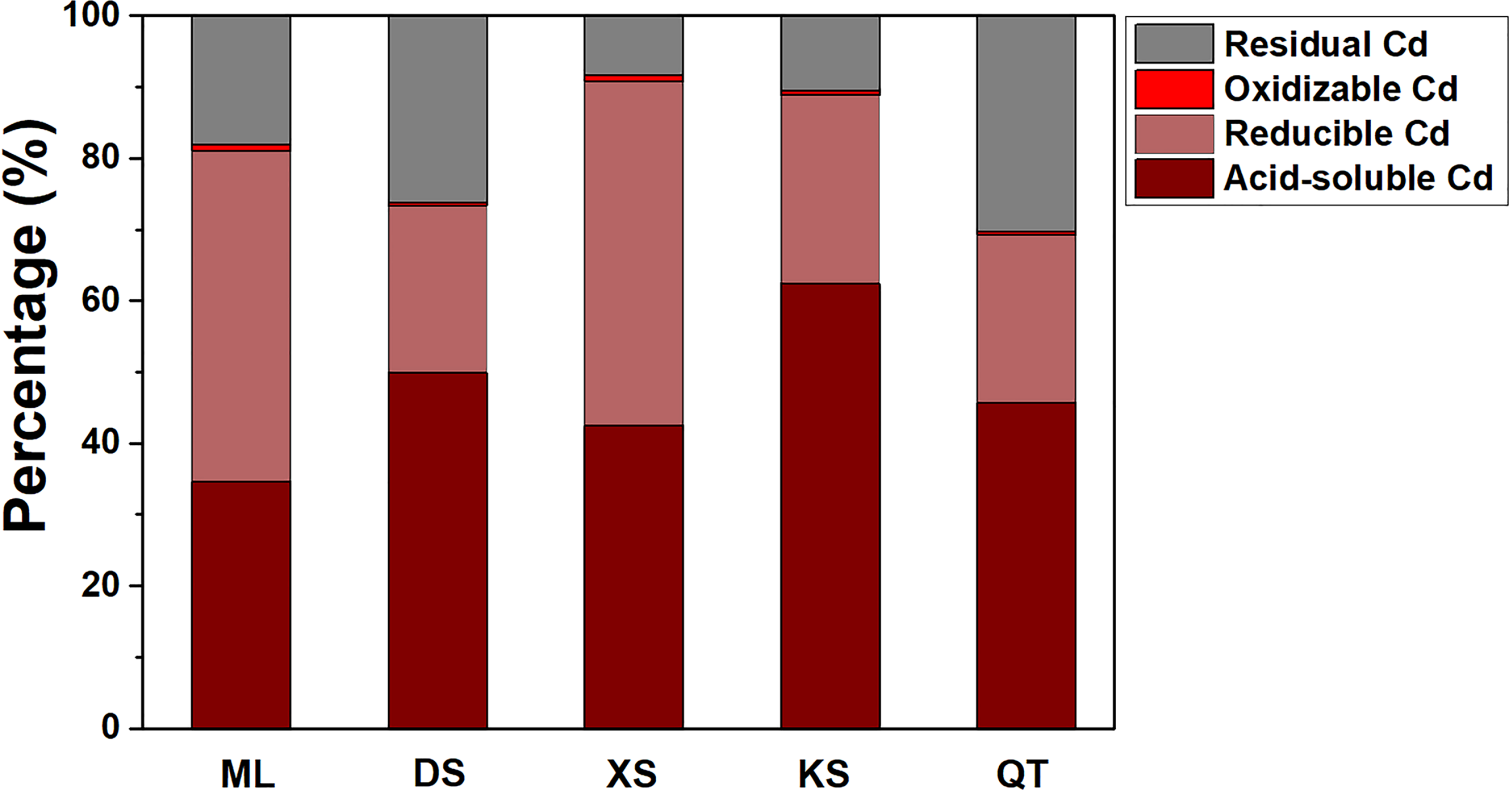
Proportions of various Cd fractions in soils sampled from five apple orchards subjected to 500 μM CdCl_2_ for 70 d. Data are means ± SE (n = 3). ML, Maliangou village in Liaoyang city; DS, Desheng village in Panjin city; XS, Xishan village in Chaoyang city; KS, Kaoshantun village in Xinmin city; QT, Qianertaizi village in Xinmin city.

The proportions of acid-soluble and reducible Cd were highest in all five soils followed by residual and oxidizable Cd (**Figure 2**). The proportions of acid-soluble Cd were significantly lower in soils of ML and XS than soils of DS and KS. By contrast, the proportions of reducible and oxidizable Cd were markedly higher in soils of ML and XS than the other soils. The percentages of residual Cd were highest in soils of DS and QT (**Figure 2**).

### 3.3 Plant Cd concentration, total Cd and BCF

**Figure 3:**
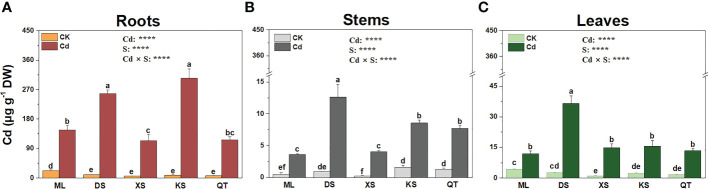
Cd concentrations in roots **(A)**, stems **(B)**, and leaves **(C)** of ‘Hanfu’ apple plants grown in soils sampled from five apple orchards subjected to 0 **(CK)** or 500 μM CdCl_2_ **(Cd)** for 70 d. Data are means ± SE (n = 3). Different letters on bars indicate significant differences between treatments. *P*-values indicated for ANOVA of Cd treatment (Cd), soil (S), and their interaction (Cd × S). ^****^ *P* < 0.0001. ML, Maliangou village in Liaoyang city; DS, Desheng village in Panjin city; XS, Xishan village in Chaoyang city; KS, Kaoshantun village in Xinmin city; QT, Qianertaizi village in Xinmin city.

Under Cd stress, the roots of the plants grown in soils of DS and KS accumulated more Cd than those of the plants grown in soils of ML, XS, and QT (**Figure 3A**). The Cd concentrations in the stems of the plants grown in soils of ML and XS were significantly lower than those of the plants grown in the other three soils (**Figure 3B**). The Cd concentrations were 135.5–212.5% higher in the leaves of the plants grown in soil of DS than they were in those of the plants grown in the other four soils (**Figure 3C**).

**Figure 4:**
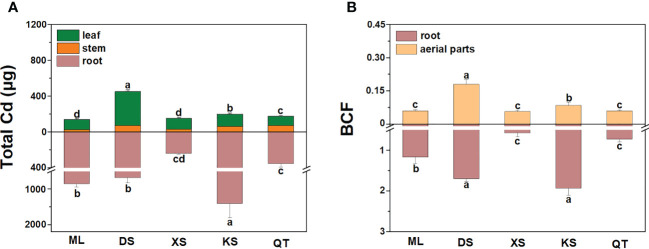
Total plant Cd content **(A)** and bio-concentration factor **(**BCF, **B)** of roots and aerial parts of ‘Hanfu’ apple plants grown in soils sampled from five apple orchards subjected to 500 μM CdCl_2_ for 70 d. Data are means ± SE (n = 3). Different letters on bars indicate significant differences between soils. ML, Maliangou village in Liaoyang city. DS, Desheng village in Panjin city; XS, Xishan village in Chaoyang city; KS, Kaoshantun village in Xinmin city; QT, Qianertaizi village in Xinmin city.

Under Cd stress, the total Cd was highest in the roots of the plants grown in soil of KS and lowest in those of the plants grown in soil of XS. The total Cd was significantly lower in the aerial parts of the plants grown in soils of ML and XS than those of the plants grown in other soils (**Figure 4A**). After Cd exposure for 70 d, the BCFs were markedly higher in the roots and aerial parts of the plants grown in soils of DS and KS than they were in those of the plants grown in soils of ML, XS, and QT (**Figure 4B**).

### 3.4 Coefficients of correlation between content of different forms of Cd and soil physicochemical properties, and Cd content in various ‘Hanfu’ apple plant tissues

Acid-soluble Cd was significantly negatively correlated with CEC and silt content but significantly positively correlated with sand content (**Table S2**). Reducible and oxidizable Cd was significantly positively correlated with OM content, CEC, and clay and silt content but significantly negatively correlated with sand content (**Table S2**). Oxidizable Cd was significantly positively correlated with pH value (**Table S2**). Residual Cd was significantly positively correlated with pH value and sand content but significantly negatively correlated with OM content, CEC, and content of clay and silt (**Table S2**). In general, root, stem and leaf Cd concentrations of apple plants were significantly positively correlated with acid-soluble Cd but negatively correlated with reducible and oxidizable Cd (**Table S3**).

### 3.5 Influences of Cd stress on growth characteristics of apple plants grown in different soils

Relative to the untreated control, Cd stress markedly reduced the dry mass of root, stem, and leaf of the plants grown in all five soils (**Table 2**). Whereas the root dry mass of the plants grown in soils of ML and XS was only slightly inhibited, the root dry mass of the plants grown in soil of DS was inhibited to the greatest extent (**Table 2**). There were no marked differences in the inhibition degree of Cd stress on stem dry mass of plants grown in different soil types. After Cd exposure, foliar biomass was inhibited in all plants but to relatively lesser degree in those grown in soils of ML and KS (**Table 2**).

**Table 2:** Dry mass (g) of roots, stems, and leaves and lengths of stems of ‘Hanfu’ apple plants grown in soils sampled from five apple orchards subjected to 0 or 500 μM CdCl_2_ for 70 d.

Soils	Cd (μM)	Root (g DW)	Stem (g DW)	Leaf (g DW)	Stem (cm)
ML	0	5.97 ± 0.34 a	7.85 ± 0.17 b	9.66 ± 0.20 b	60.50 ± 1.59 bc
	500	4.58 ± 0.06 c	5.16 ± 0.21 d	8.60 ± 0.173 d	53.88 ± 0.07 d
DS	0	3.02 ± 0.26 ef	6.43 ± 0.11 c	9.48 ± 0.28 bc	55.13 ± 1.37 d
	500	1.27 ± 0.144 g	4.10 ± 0.245 e	7.41 ± 0.15 ef	45.13 ± 0.51 e
XS	0	3.32 ± 0.08 de	7.32 ± 0.193 b	9.14 ± 0.02 bcd	72.78 ± 0.66 a
	500	2.68 ± 0.14 f	4.50 ± 0.10 e	6.92 ± 0.32 f	61.50 ± 2.50 bc
KS	0	5.44 ± 0.10 b	7.21 ± 0.45 b	7.66 ± 0.23 e	64.50 ± 1.59 b
	500	3.59 ± 0.05 d	4.74 ± 0.08 de	6.94 ± 0.12 f	54.88 ± 0.07 d
QT	0	5.17 ± 0.19 b	8.96 ± 0.24 a	10.96 ± 0.42 a	63.50 ± 3.03 b
	500	3.07 ± 0.08 ef	6.12 ± 0.15 c	8.85 ± 0.31 cd	57.00 ± 0.87 cd
*P*-value	Cd	^****^	^****^	^****^	^****^
	S	^****^	^****^	^****^	^****^
	Cd×S	^*^	ns	^*^	ns

Data are means ± SE (n = 3). Different letters beside values indicate significant differences between treatments. *P*-values indicated for ANOVA of Cd treatment (Cd), soil (S), and their interaction (Cd × S). ^*^ *P* < 0.05. ^****^ *P* < 0.0001. ns, not significant. ML, Maliangou village in Liaoyang city; DS, Desheng village in Panjin city; XS, Xishan village in Chaoyang city; KS, Kaoshantun village in Xinmin city; QT, Qianertaizi village in Xinmin city.

The total root length, total root surface area, and total root volume of the ‘Hanfu’ plants significantly decreased after Cd stress. However, these negative effects were relatively less pronounced in the plants grown in soil of ML and relatively more pronounced in those grown in soil of DS (**Table S4**). The chlorophyll content considerably differed among the plants grown in different soils (**Table S4**). Cd stress reduced chlorophylls a, b, (a+b), and carotenoid in all plants but to a greater extent in those grown in soil of KS than the others (**Table S4**).

### 3.6 Effects of Cd stress on O_2_ ^·−^, H_2_O_2_, and MDA in plants grown under different soil conditions

**Figure 5:**
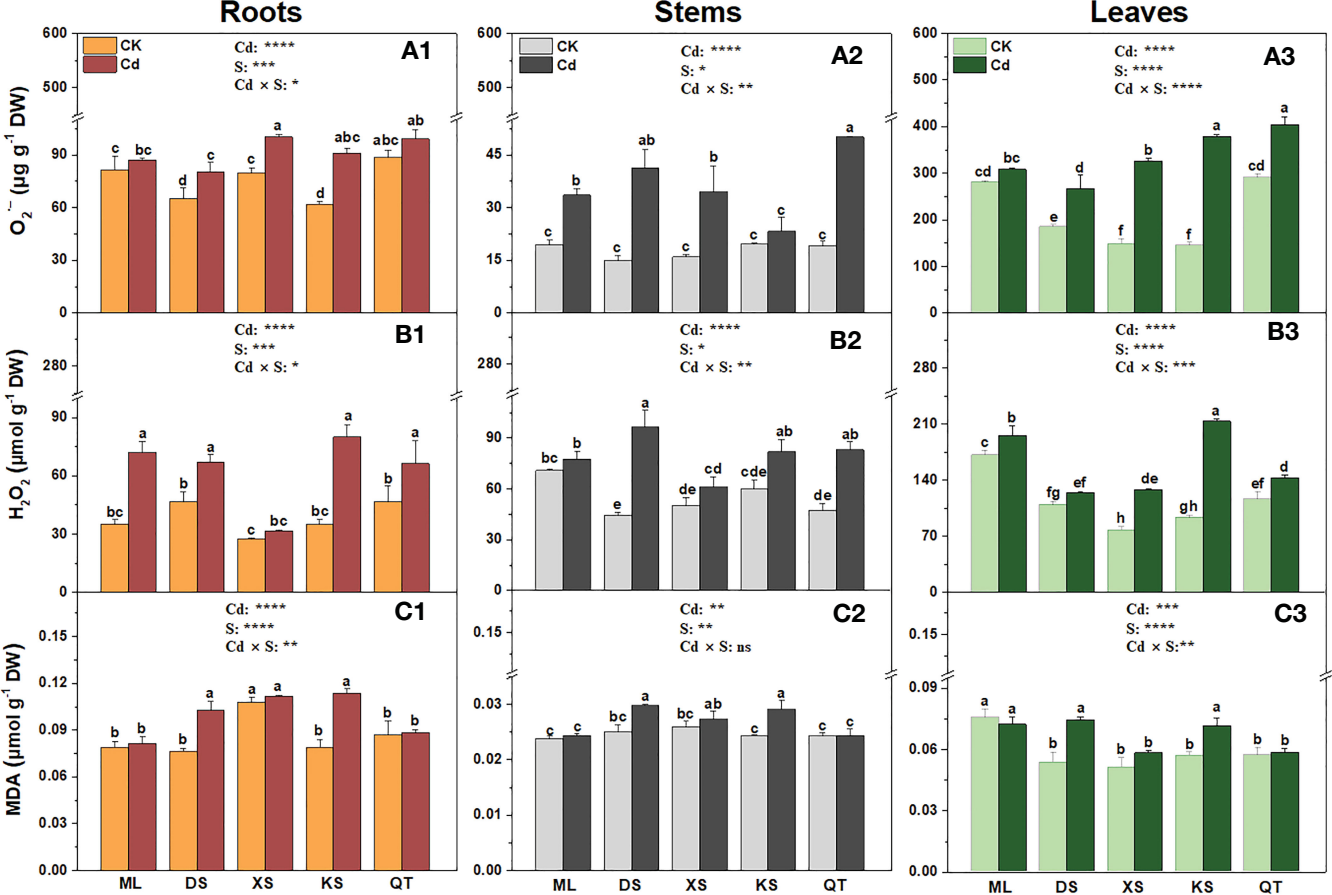
Superoxide anion ( ${\text{O}}_{2}^{\;\cdot -}$ , **A1-A3**), hydrogen peroxide (H_2_O_2_, **B1-B3**) and malondialdehyde (MDA, **C1-C3**) in roots, stems and leaves of ‘Hanfu’ apple plants grown in soils sampled from five apple orchards subjected 0 (CK) or 500 μM CdCl_2_ (Cd) for 70 d. Data are means ± SE (n = 3). Different letters on bars indicate significant differences between treatments. *P*-values indicated for ANOVA of Cd treatment (Cd), soil (S), and their interaction (Cd × S). *P < 0.05. **P < 0.01. ***P < 0.001. ****P < 0.0001. ns, not significant. ML, Maliangou village in Liaoyang city; DS, Desheng village in Panjin city; XS, Xishan village in Chaoyang city; KS, Kaoshantun village in Xinmin city; QT, Qianertaizi village in Xinmin city.

Cd exposure significantly increased ${\text{O}}_{2}^{\;\cdot -}$ accumulation in the roots of the plants grown in soils of DS, XS and KS (**Figure 5**). Cd exposure induced a more pronounced increase in ${\text{O}}_{2}^{\;\cdot -}$ concentrations in the stems of plants grown in the soils of DS and QT (**Figure 5**). After Cd exposure, the ${\text{O}}_{2}^{\;\cdot -}$ concentrations were significantly increased in the leaves of the plants grown in all soils except for soil of ML (**Figure 5**). Cd exposure significantly increased the H_2_O_2_ content in the roots of all plants except those grown in soil of XS, in the stems of all plants except those grown in soils of ML and XS, and in the leaves of all plants except those grown in soil of DS (**Figure 5**). Cd induced H_2_O_2_ accumulation to the greatest extent in the roots and leaves of the plants grown in soil of KS and in the stems of the plants grown in soil of DS (**Figure 5**). Compared with the control, Cd exposure had no effects on the MDA concentration in the roots, stems, or leaves of the plants grown in soils of ML, XS, and QT but significantly increased it in the same organs of the plants grown in soils of DS and KS (**Figure 5**).

### 3.7 Effects of Cd stress on the antioxidant systems of plants grown in different soils

**Figure 6:**
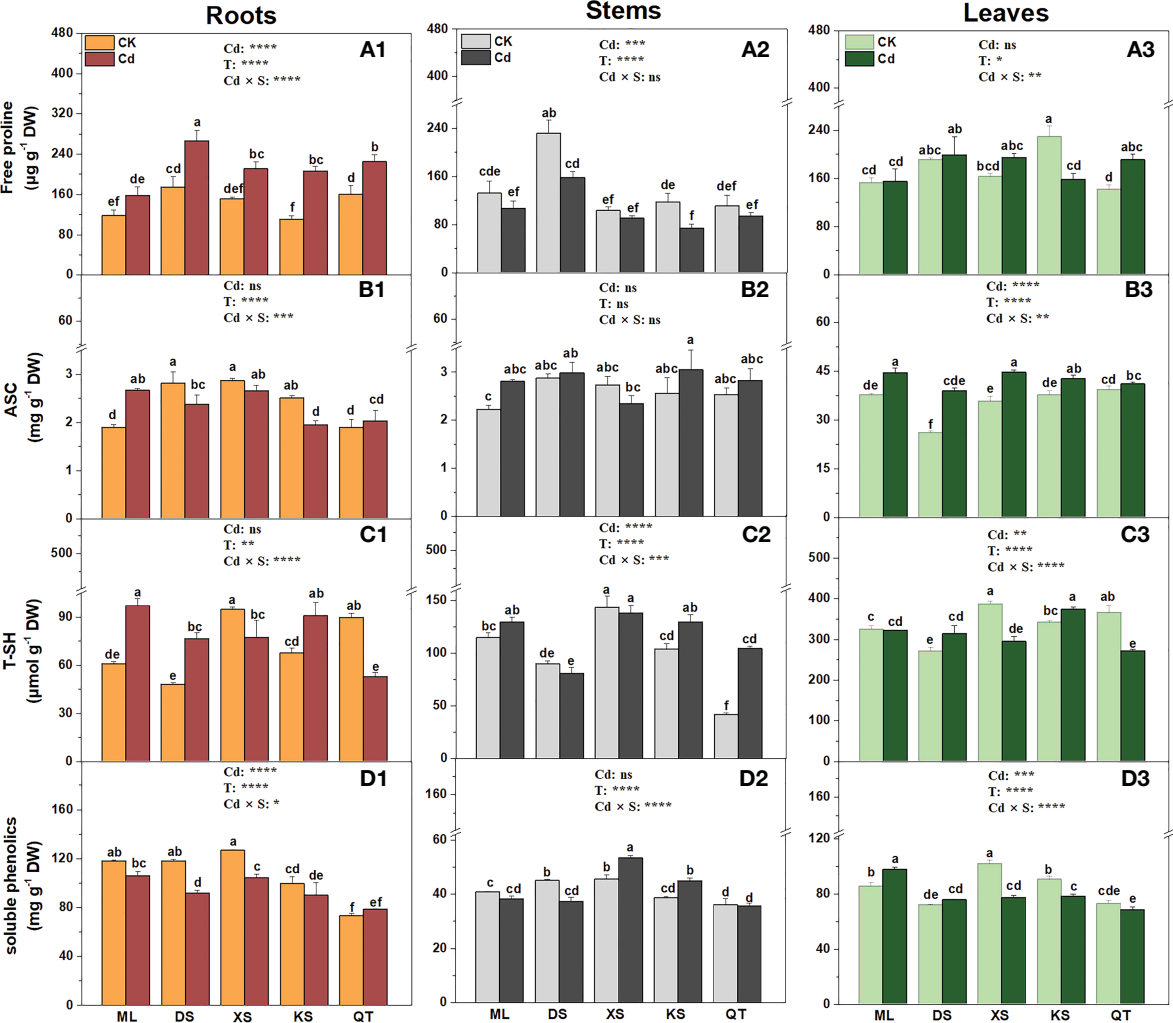
Free proline **(A1-A3)**, ascorbate (ASC) (**B1-B3**), total thiols (T-SH) **(C1-C3)** and soluble phenolics **(D1-D3)** in the roots **(A1-D1)**, stems **(A2-D2)** and leaves **(A3-D3)** of ‘Hanfu’ apple plants grown in the soils sampled from five apple orchards subjected to 0 (CK) or 500 μM CdCl_2_ (Cd) for 70 d. Data indicate means ± SE (n = 3). Different letters on the bars indicate significant differences between the treatments. *P*-values indicated for ANOVA of Cd treatment (Cd), soil (S) and their interaction (Cd × S). *P < 0.05. **P < 0.01. ***P <0.001. ****P < 0.0001. ns, not significant. ML, Maliangou village in Liaoyang city; DS, Desheng village in Panjin city; XS, Xishan village in Chaoyang city; KS, Kaoshantun cillage in Xinmin city; QT, Qianertaizi village in Xinmin city.

Compared to the control, Cd stress significantly increased the free proline content in the roots of the plants grown in all soils except soil of ML (**Figure 6A1**). Cd exposure significantly lowered the free proline concentration in the stems of the plants grown in soils of DS and KS and in the leaves of the plants grown in soil of KS (**Figures 6A2, A3**). After Cd exposure, the ASC content increased in the roots and stems of the plants grown in soil of ML and the leaves of the plants grown in all soils except for soil of QT. However, it decreased in the roots of the plants grown in soils of DS and KS (**Figures 6B1–B3**). Under Cd stress, the T-SH content was highest in the roots of the plants grown in soil of ML, the stems of the plants grown in soil of XS, the leaves of the plants grown in soil of KS (**Figures 6C1–C3**). Cd exposure significantly decreased the soluble phenolic content in the roots of the plants grown in soils of DS and XS, but slightly increased the soluble phenolic content in the stems of the plants grown in soils of XS and KS and the leaves of the plants grown in soil of ML (**Figures 6D1–D3**).

After Cd exposure, the SOD was significantly enhanced by 98.7%, 344.5%, and 105.9% in the roots of the plants grown in soils of DS, XS, and KS, respectively (**Figure S1A1**). In general, Cd exposure increased SOD activity in the stems and leaves of all plants except the leaves of plants grown in soil of XS and QT (**Figures S1A2, A3**). Irrespective of Cd treatment, SOD activity was always higher in the stems of the plants grown in soil of ML than it was in those of the plants grown in all other soils (**Figure S1A2**). Generally, Cd exposure increased POD activity in all tissues of the plants grown in most soils compared to those under control conditions (**Figures S1B1–B3**). The highest POD activity was observed in the roots of the plants grown in soil of QT and the stems of the plants grown in soil of ML (**Figures S1B1, B2**). Cd exposure generally increased CAT in the roots and stems of most plants especially in the stems of the plants grown in soil of XS (**Figures S1C1, C2**). By contrast, CAT activity increased only in the leaves of the plants grown in soil of DS (**Figure S1C3**). Compared with the control, APX activity was elevated in the roots of all plants except those grown in soil of KS (**Figure S1D1**). By contrast, APX activity was only increased in the stems of the plants grown in soils of DS and QT. The leaves of the plants grown in all five soil types did not differ in terms of APX activity following Cd exposure (**Figures S1D2, D3**). GR activity increased only in the roots of the plants grown in soil of QT, the stems of the plants grown in soils of ML and XS, and the leaves of the plants grown in soil of DS (**Figures S1E1–E3**).

### 3.8 PCA of physiological responses to Cd in apple plants grown under different soil conditions

**Figure 7:**
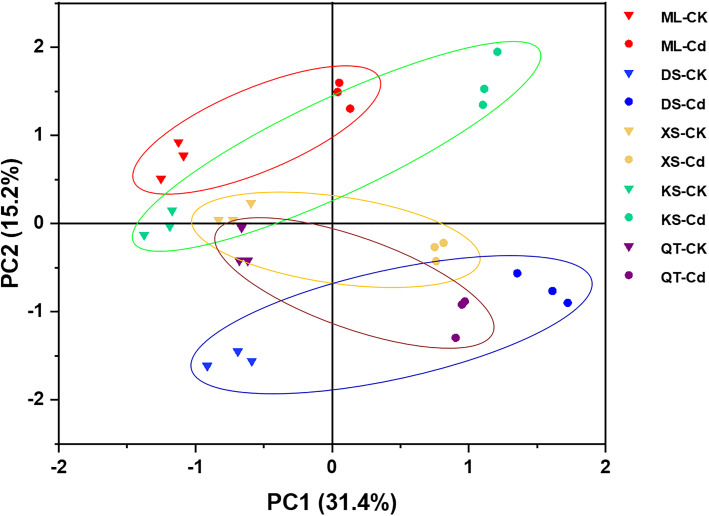
Principal component analysis (PCA) of plant growth related parameters, photosynthetic pigments, Cd concentrations, ROS and antioxidant levels in roots, stems, and leaves of apple plants grown in soils sampled from five apple orchards subjected to 0 (CK) or 500 μM CdCl_2_ (Cd) for 70 d. Triangles and circles represent 0 (CK) and 500 μM CdCl_2_ (Cd) treatments, respectively. Different colors represent different soils. Red, Maliangou village in Liaoyang city. Blue, Desheng village in Panjin city. Yellow, Xishan village in Chaoyang city. Green, Kaoshantun village in Xinmin city. Purple, Qianertaizi village in Xinmin city. PCA loadings presented in **Table S5**.

To elucidate the response patterns of ‘Hanfu’ apple plants to Cd stress, a PCA was conducted using data associated with plant growth, photosynthetic pigments, Cd concentrations, and the ROS and antioxidant levels in the roots, stems, and leaves (**Figure 7**; **Table S5**). The effect of Cd treatment was separated by PC1 and its contribution rate was 31.4% (**Figure 7**). The dominant components in PC1 were root Cd concentration and free proline content, stem dry mass and Cd content, and leaf Cd content (**Table S5**). However, PC2 was affected by soil and its contribution rate was 15.2% (**Figure 7**). The dominant factors in PC2 were root POD and GR content, stem SOD content, and leaf H_2_O_2_ and SOD content (**Table S5**). The PCA showed that plants grown in various soils demonstrated different physiological responses to Cd exposure mainly because of the relative differences in their root POD and GR content, their stem SOD content, and their leaf H_2_O_2_ and SOD content. In the PCA plot, the shorter distances between CK and Cd treatment in soils of ML and XS indicated that the plants grown in these soils were relatively less affected by Cd exposure at the physiological level.

### 3.9 Transcriptional changes of the genes involved in Cd absorption, transport, and detoxification

**Figure 8:**
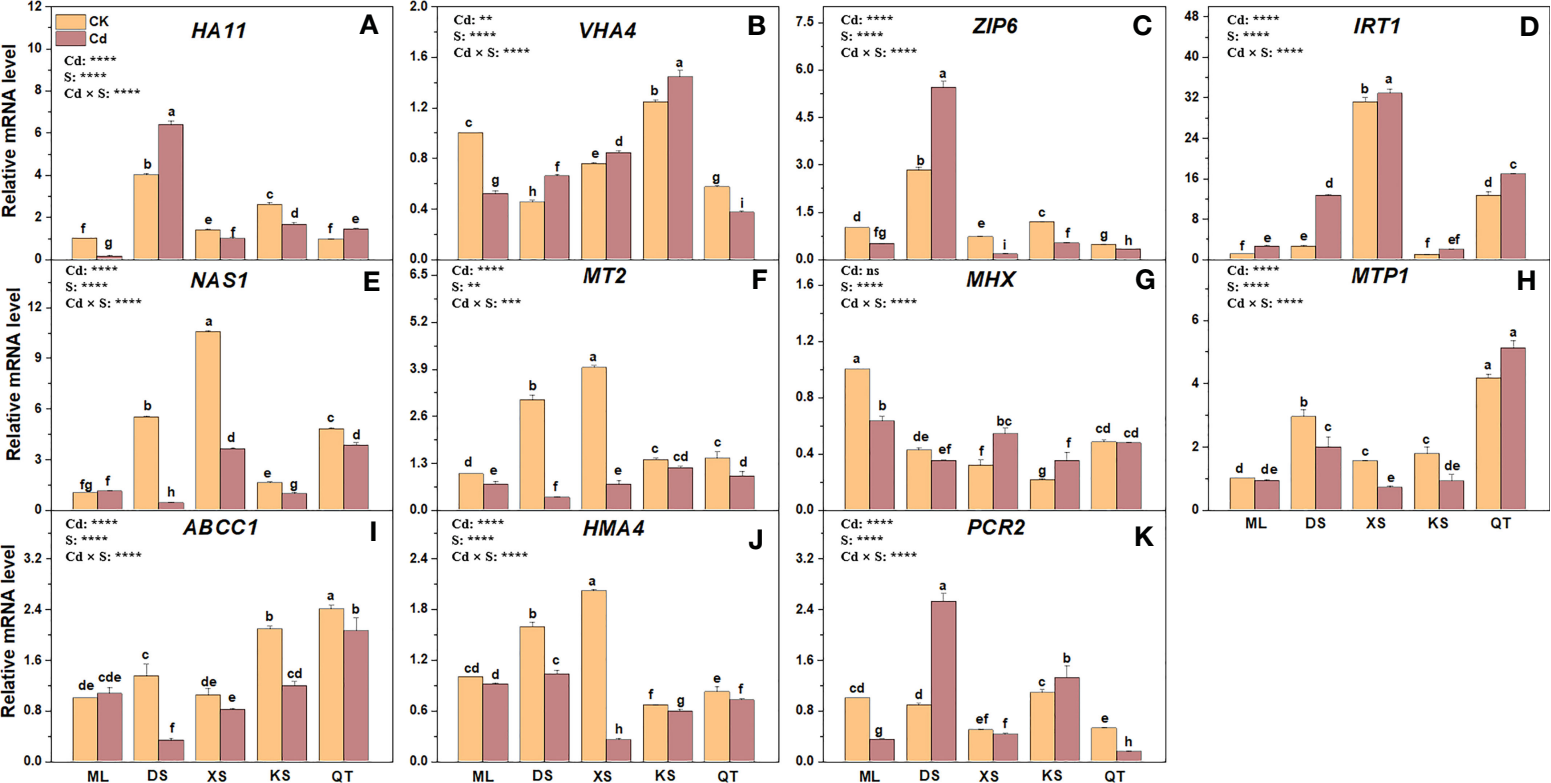
Relative expression levels of genes encoding proteins regulating Cd absorption **(A–D)** detoxification **(E–I)** and transport **(J, K)** in roots of ‘Hanfu’ apple plants grown in soils sampled from five apple orchards subjected to 0 (CK) or 500 μM CdCl_2_ (Cd) for 70 d. Data are means ± SE (n = 3). Different letters on the bars indicate significant differences between the treatments. For each gene, expression level was normalized to the reference gene *β-Actin* and the expression level was set to 1 in the roots of ‘Hanfu’ apple plants grown in soil of ML subjected to 0 μM CdCl_2_. Corresponding fold changes in other treatments were then calculated accordingly. *P*-values indicated for ANOVA of Cd treatment (Cd), soil (S) and their interaction (Cd × S). **P < 0.01. ***P < 0.001. ****P < 0.0001. ns, not significant. ML, Maliangou village in Liaoyang city; DS, Desheng village in Panjin city; XS, Xishan village in Chaoyang city; KS, Kaoshantun village in Xinmin city; QT, Qianertaizi village in Xinmin city.

PM H^+^-ATPase pumps protons out of cells by consuming ATP, creates an electrochemical gradient for transmembrane ion transport (Zhang et al., 2017), and is encoded by several genes including *HA11* and *VHA4*. Cd exposure downregulated *HA11* in the roots of the plants grown in soils of ML, XS, and KS by 7.14-fold, 1.43-fold, and 1.58-fold, respectively (**Figure 8**). By contrast, Cd exposure upregulated *HA11* in the roots of the plants grown in soils of DS and QT, relative to their respective controls (**Figure 8**). Compared to untreated controls, the roots of the plants grown in soils of ML and QT presented with *VHA4* downregulation. However, the opposite trend was observed in the roots of the plants grown in all other soils. *ZIP* and *IRT1* transport Cd^2+^ intracellularly and regulate plant Cd absorption (Migeon et al., 2010; Wu et al., 2015). Compared with the control, Cd exposure upregulated *ZIP6* in the roots of the plants grown in soil of DS but downregulated it in the roots of the plants grown in all other soils and especially those of the plants grown in soil of XS. Cd exposure upregulated *IRT1* in the roots of the plants grown in all soils but especially in those of the plants grown in soil of DS (**Figure 8**).

*NAS1* and *MT2* may regulate Cd detoxification in plants (Luo et al., 2016). Cd exposure did not affect *NAS1* expression in the roots of the plants grown in soil of ML but markedly downregulated *NAS1* in those of the plants grown in the other soils and especially soil of DS (**Figure 8**). Cd exposure downregulated *MT2* in the roots of the plants grown in all soils and especially in those of the plants grown in soil of DS (**Figure 8**). *MHX*, *MTP1* and *ABCC1* are localized to the tonoplast and transfer Cd^2+^ or the Cd-PC complex to the vacuole for storage (Berezin et al., 2008; Lin and Aarts, 2012; Park et al., 2012). Compared with the control, Cd exposure upregulated *MHX* only in the roots of the plants grown in soils of XS and KS. Relative to the control, Cd exposure markedly downregulated *MTP1* in the roots of all plants except those grown in soils of ML and QT (**Figure 8**). Compared with the control, Cd exposure downregulated *ABCC1* in the roots of the plants grown in soils of DS and KS but not in the roots of the plants grown in soils of ML and XS (**Figure 8**).

PM-bound transporters such as HMA4 and PCR2 export Cd from the cytosol, facilitating Cd translocation into the central cylinder for xylem transport (Hanikenne et al., 2008; Song et al., 2010). Cd treatment caused downregulation of *HMA4* in the roots of the plants grown in all soils but to a greater extent in the roots of those grown in soil of XS (**Figure 8**). After Cd exposure, *PCR2* was downregulated in the roots of the plants grown in soils of ML and QT but upregulated in the roots of the plants grown in soils of DS and KS (**Figure 8**).

## 4 Discussion

### 4.1 Variations in Cd accumulation and tolerance in apple plants grown in different soils

Heavy metals in soils are compartmentalized into the acid-soluble, reducible, oxidizable, and residual fractions (Meng et al., 2018). Of these, the acid-soluble fraction has the highest mobility (Matong et al., 2016; Zhang et al., 2018b). Soil Cd fractions with different mobility are readily affected by the soil physicochemical properties (Mohamed et al., 2015; Filipović et al., 2018; Xiao et al., 2019). Elevated OM content, CEC, and silty clay loam content provide abundant exchange sites for Cd adsorption, reduce the soil available Cd content, and lower plant Cd uptake (Mohamed et al., 2015; Filipović et al., 2018). In contrast, high soil sand content decreases the surface area and the total negative charge and is not, therefore, conducive to metal cation retention in the soil (Sungur et al., 2014a). Consistent with these results, higher OM, CEC, silt content and lower sand content in soils of ML and XS led to relatively lower acid-soluble Cd and higher reducible and oxidizable Cd content and proportions. He et al. (2017) found that soil Cd availability decreased with the increase of soil pH value. Soil of KS had the lowest soil pH, which may be an important reason for the higher acid-soluble Cd content in soil of KS. Interestingly, the correlations between pH value and acid-soluble and reducible Cd were not significant, indicating that pH value played less important roles in regulating soil Cd forms than other soil characteristics in the present study.

Plant Cd content is usually positively correlated with soil bioavailable Cd (Geebelen et al., 2003). In this study, Cd accumulation varied greatly in apple plants grown in different soils. Plants grown in soils of ML and XS presented with lower root and stem Cd accumulation and BCF, which is attributed to relatively lower Cd bioavailability in these soils. Biomass, root architecture, and plant pigment content reflect heavy metal phytotoxicity (Shi and Cai, 2009). Plants grown in different soils with distinct physicochemical properties varied greatly in their growth parameters (Patel et al., 2015). Apple plants grown in soils ML and XS displayed lower inhibition of tissue biomass and root architecture as well as photosynthetic pigment, while the corresponding indexes of soils of DS, KS and QT showed opposite results. Thus, the plants grown in soils of ML and XS have a greater Cd tolerance as these substrates had relatively higher OM content, CEC, and silt content, lower sand content, and, therefore, low Cd mobility. These results are consistent with those reported in cabbage and maize under Cd stress (Mohamed et al., 2015).

### 4.2 Physiological basis of the differences in Cd tolerance among plants grown under various soil conditions

Cd stress induces the accumulation of ROS and MDA, therefore, lead to oxidative stress in plants (Muradoglu et al., 2015). Here, the reductions in growth and photosynthetic pigment content of plant grown in five different soils under Cd stress are associated with Cd-induced oxidative stress. However, as the Cd accumulation was relatively lower in the plants grown in soils of ML and XS, there was relatively less oxidative stress in the plants grown in these two soils, indicating that these plants may adopt more effective coordinated regulatory mechanisms than the plants grown in the other soils.

Cd stress induces plants to produce non-enzymatic antioxidants, for example free proline, ASC, soluble phenolics, and T-SH to remove ROS and mitigate Cd phytotoxicity (He et al., 2020). Hussain et al. (2022) found that biochar application to wheat (*Triticum aestivum* L.) plants under Cd stress increased their foliar free proline, and total soluble phenolics content, which may be attributed to high pH and CEC of biochar (Cui et al., 2016; Moradi et al., 2019). Here, relative higher levels of non-enzymatic antioxidants in plants grown in soils of ML and XS are probably contributed to lower ROS and MDA accumulation. SOD, POD, CAT, APX, and GR are key antioxidant enzymes, playing pivotal roles in overcoming Cd-induced oxidative stress in plants. Biochar amendment significantly increased these enzyme activities in the leaves of pak choi (*Brassica chinensis* L.) under Cd stress possibly because biochar alters soil physicochemical properties (Kamran et al., 2019). Under Cd treatment, the lower ROS and MDA levels in the plants grown in soils of ML and XS were associated with increased antioxidant enzymes activities in tissues of plants grown in these two soils.

### 4.3 Variations in Cd accumulation and tolerance are related to different gene expression patterns in apple plants grown under various soil conditions

Our previous study showed that PM H^+^-ATPase gene downregulation inhibited Cd uptake by apple rootstocks (He et al., 2020) and that PM H^+^-ATPase activity might be affected by soil physicochemical properties (Zhang et al., 2018a; Olaetxea et al., 2019). Under Cd exposure conditions, the expression levels of *HA11* in the plants grown in soils of ML and XS and the expression level of *VHA4* in the plants grown in soil of ML were lower than those in the plants grown in soils of DS and KS. These observations were consistent with the fact that Cd concentrations were relatively lower in the roots of the plants grown in soils of ML and XS. *ZIP* and *IRT1* regulated Cd absorption in *Populus* × *canescens* and Chinese cabbage, respectively (Wu et al., 2015; Ding et al., 2017). Nevertheless, there is little empirical evidence about the expression of *ZIP* and *IRT1* regulating Cd uptake in plants grown under different soil conditions. Here, the downregulation of *ZIP6* in the plants grown in soils of ML and XS, and the upregulation of *ZIP* and *IRT1* in the plants grown in soil of DS were consistent with root Cd concentration in these plants. Therefore, variations in Cd accumulation are related to differential transcriptional regulation of genes related to Cd uptake in apple plants grown in different soils.

*Arabidopsis thaliana* harboring overexpressing *Brassica juncea* gene encoding MT2 had lower Cd sensitivity and higher Cd tolerance than its wild type counterpart (Zhigang et al., 2006). Under Cd stress, three *NAS* genes were strongly upregulated in the roots of durum wheat, thereby maintaining NA production and lowering Cd phytotoxicity (Aprile et al., 2018). Jaskulak et al. (2019) found that soil and metal cation type affected *MT* expression. In this study, the lowest mRNA levels of *MT2* and *NAS1* were found in the plants grown in soil of DS subjected to Cd stress. These findings were in line with the fact that Cd phytotoxicity was more severe in the plants grown in soil of DS. By contrast, the less repression of transcript levels of *MT2* in plants grown in soil of ML, and *NAS1* in plants grown in soils of ML and XS by Cd than in soil of DS corresponding well to its relative lower Cd toxicity than those grown in the soils of DS.

Transporters such as MHX, MTP1, and ABCC1 are located on the tonoplast, transport Cd or Cd complexes from the cytoplasm to vacuole, reduce cytoplasmic free Cd content (Berezin et al., 2008; Lin and Aarts, 2012), and mitigate Cd phytotoxicity. However, there is little empirical evidence that soil physicochemical properties affect transcription of the genes regulating Cd detoxification. Only Jaskulak et al. (2020) found that relative expression of *ABCC* in the shoots of *Sinapis alba* L. varied with soil CEC and total organic carbon content. We found that *MHX* was relatively upregulated in the roots of the plants grown in soil of XS subjected to Cd stress. Cd exposure downregulated *MTP1* in the roots of the plants grown in all soils except in soils of ML and QT and downregulated *ABCC1* in roots of the plants grown in soils of DS and KS. The foregoing results might indicate that the plants grown in soils of ML and XS had relatively lower Cd toxicity as they sequestered the Cd in vacuoles or formed relatively nontoxic Cd-containing complexes.

HMA4 and PCR2 are localized to the plasma membrane and transport Cd^2+^ extracellularly (Hanikenne et al., 2008; Song et al., 2010). Our previous study showed that exogenous melatonin restricted root-to-shoot Cd transport in apple rootstock by downregulating root *HMA4* and *PCR2* (He et al., 2020). In the present study, compared with soils of DS and KS, the *HMA4* levels were relatively lower in the roots of the plants grown in soil of XS and the *PCR2* levels were relatively lower in the roots of the plants grown in soils of ML, XS, and QT under Cd stress. For these reasons, these plants effectively inhibited Cd root-to-shoot transport and mitigated its shoot toxicity.

## 5 Conclusions

There were significant differences in Cd accumulation, translocation and tolerance in apple plants grown in soils with different physicochemical properties. Lower Cd accumulation and BCF were found in apple plants grown in soils of ML and XS, which could be ascribed to lower Cd bioavailability in these soils. The negative effects of Cd stress on the growth were less pronounced in the plants grown in soils of ML and XS. Moreover, the plants grown in these two soils had relatively lower ROS and MDA production, but higher antioxidant content and enzyme activity. The expression of genes related to Cd absorption and transport was lower in the roots of plants grown in the soils of ML and XS while the transcription levels of the key genes related to Cd detoxification were higher. Our data demonstrated that apple plants grown in soils of ML and XS with higher OM content, CEC, clay and silt content and lower sand content accumulated less Cd and had relative higher Cd tolerance, indicating that the soil types affect Cd accumulation and tolerance in apple plants. These results lay the foundation for mitigation strategies by application of amendments to modify soil properties to decrease soil Cd bioavailability.

## Data availability statement

The raw data supporting the conclusions of this article will be made available by the authors, without undue reservation.

## Author contributions

XZ and JH conceived and designed research. XZ, HXW and HYW performed the experiment. XZ and JH analyzed data and prepared the manuscript. JH, SQ and DL are responsible for conceptualization, funding acquisition, review and editing. All authors read and approved the final manuscript.
