# Supplementary material for: Characteristics of cadmium accumulation and tolerance in apple plants grown in different soils
Source: Front Plant Sci. 2023 Jun 2;14:1188241. doi: 10.3389/fpls.2023.1188241 (PMC10272767; doi:10.3389/fpls.2023.1188241)

Supplementary Material

Characteristics of cadmium accumulation and tolerance in apple plants grown in different soils

Xiaolei Zhuang^1,2^, Huixue Wan^1,2^, Hongyu Wang^1^, Sijun Qin^1,2^, Jiali He^1,2*^, Deguo Lyu^1,2*^

*** Correspondence:
Name:** Jiali He, Deguo Lyu

**Tel:** 0086-24-88487143
**E-mail:** Jiali He (hejiali1017@163.com; hejiali1017@syau.edu.cn), Deguo Lyu (lvdeguo@163.com)

**Table S1** Primers used for qRT-PCR.

| Gene name | Description | Primer-Forward | Primer-Reverse |
| --- | --- | --- | --- |
| *HA11* ^a^ | PM H^+^-ATPase | 5’-TGCTTTGCTAGGACAGGACA-3’ | 5’-GAAGCACTACGAGCAGCATC-3’ |
| *VHA4* ^a^ | PM H^+^-ATPase | 5’-GATGGTGTCAATGATGCCCC-3’ | 5’-ACACTCAATCCGGGTTCTGT-3’ |
| *ZIP6* ^a^ | zinc transporter | 5’-CGGTGGGTGTGGAGAGTAAT-3’ | 5’-ATGTAGGCTGTTGTCCCGAA-3’ |
| *IRT1* ^b^ | iron transporter | 5’-TTGACAAGGGAGAAAACGGAGAC-3’ | 5’-AACAACTGAATGGACAATGATACCC-3’ |
| *NAS1* ^b^ | nicotianamine synthase | 5’-CGATGTTTCCAAGTTATGCCAA-3’ | 5’-TTCTCCAAAAGTCCTTCTGCCT-3’ |
| *MT2* ^c^ | metallothionein | 5’-CAACGGGTGCGGGATGGCTCCTGAT-3’ | 5’-GCAGGGGTTGCAGGTGCAGCTATCC-3’ |
| *MHX* ^a^ | magnesium proton exchanger protein | 5’-TCAAGTTAGAAGACACGGAATC-3’ | 5’-AGGCACAAATGCGAATAG-3’ |
| *MTP1* ^a^ | metal tolerance protein | 5’-CGACAAGGATCTTGCTGAGC-3’ | 5’-TCCCCAGAACAATGACCGAA-3’ |
| *ABCC1* ^a^ | ABC transporter C | 5’-TGATGATCCCTTGAGTGC-3’ | 5’-TTTTGAACAGCGTGCC-3’ |
| *HMA4* ^a^ | P-type heavy metal ATPase | 5’-GGCATCCGCTCGTTT-3’ | 5’-TTTGGGGTTTACAGGCT-3’ |
| *PCR2* ^a^ | protein plant cadmium resistance | 5’-ACCACCATCACCACCAAGAT-3’ | 5’-TGGGAGCTCATGTCTTCGTT-3’ |
| *β-Actin* ^d^ |  | 5’-TACTCAGCTTTGGCAATCCACATC-3’ | 5’-TGACCGAATGAGCAAGGAAATTACT-3’ |

**Note:** a: This primer was designed by ourselves. The gene name was based on the database of phytozome *Malus domestica* v1.0. b: From Zha et al. 2014. Both immanently high active iron contents and increased root ferrous uptake in response to low iron stress contribute to the iron deficiency tolerance in *Malus xiaojinensis*. Plant Science. 214:47-56. c: From Wang et al. 2012. Cloning and expression analysis of metallothionein gene (*MhMT2*) in *Malus hupehensis*. Scientia Agricultura Sinica. 45(14):2904-2912. d: From Wang et al. 2017. MYB12 and MYB22 play essential roles in proanthocyanidin and flavonol synthesis in red-fleshed apple (*Malus sieversii f.niedzwetzkyana*). The Plant Journal, 90(2):276-292.

**Table S2** Coefficients of correlation between Cd content in different soil types and physicochemical properties of soil subjected to 500 μM CdCl_2_ for 70 d.

| Variable | pH | OM  (%) | CEC  (cmol kg^-1^) | Clay  (%) | Silt  (%) | Sand  (%) |
| --- | --- | --- | --- | --- | --- | --- |
| Acid-soluble Cd (μg g^-1^) | -0.324 | -0.507 | -0.825** | -0.410 | -0.804** | 0.793** |
| Reducible Cd (μg g^-1^) | -0.174 | 0.798** | 0.986** | 0.692** | 0.890** | -0.972** |
| Oxidizable Cd (μg g^-1^) | 0.553* | 0.732** | 0.856** | 0.517* | 0.886** | -0.900** |
| Residual Cd (μg g^-1^) | 0.647** | -0.766** | -0.651** | -0.660** | -0.522* | 0.671** |

**Note:** **P* < 0.05. ***P* < 0.01. OM, organic matter. CEC, cation exchange capacity.

**Table S3** Coefficients of correlation between Cd content in different tissues of ‘Hanfu’ apple plants and different Cd forms in soils subjected to 500 μM CdCl_2_ for 70 d.

| Variable | Acid-soluble Cd  (μg g^-1^) | Reducible Cd  (μg g^-1^) | Oxidizable Cd  (μg g^-1^) | Residual Cd  (μg g^-1^) |
| --- | --- | --- | --- | --- |
| Root Cd (μg g^-1^) | 0.761** | -0.562* | -0.214 | 0.141 |
| Stem Cd (μg g^-1^) | 0.603* | -0.873** | -0.782** | 0.734** |
| Leaf Cd (μg g^-1^) | 0.327 | -0.529* | -0.535* | 0.587* |

**Note:** ***P* < 0.05. ***P* < 0.01.

**Table S4** The total root length (m), root surface area (cm^2^), root volume (cm^3^) in roots and photosynthetic pigments (mg g^-1^ DW) in leaves of ‘Hanfu’ apple plants grown in the soils sampled from five apple orchards subjected to 0 or 500 μM CdCl_2_ treatments for 70 days.

| Soils | Cd  (μM) | Total root length  (m) | Total root surface area (cm^2^) | Total root volume (cm^3^) | Chl a  (mg g^-1^ DW) | Chl b  (mg g^-1^ DW) | Chl (a + b)  (mg g^-1^ DW) | Carotenoid  (mg g^-1^DW) |
| --- | --- | --- | --- | --- | --- | --- | --- | --- |
| ML | 0 | 212.30 ± 2.96 a | 2138.53 ± 54.67 a | 16.31 ± 1.44 ab | 5.32 ± 0.15 a | 1.55 ± 0.07 a | 6.87 ± 0.22 a | 1.15 ± 0.03 a |
|  | 500 | 171.83 ± 9.53 b | 1573.48 ± 27.80 b | 11.52 ± 0.23 cd | 4.49 ± 0.11 b | 1.35 ± 0.02 bc | 5.84 ± 0.12 b | 0.99 ± 0.01 bc |
| DS | 0 | 93.69 ± 2.00 e | 1256.66 ± 34.16 c | 14.15 ± 0.53 bc | 5.23 ± 0.28 a | 1.48 ± 0.08 ab | 6.70 ± 0.36 a | 1.10 ± 0.07 a |
|  | 500 | 44.40 ± 2.85 f | 493.27 ± 36.62 f | 5.50 ± 1.20 f | 4.43 ± 0.14 b | 1.29 ± 0.03 cd | 5.72 ± 0.17 bc | 0.92 ± 0.01 cd |
| XS | 0 | 123.80 ± 6.62 d | 1175.46 ± 68.17 cd | 9.40 ± 0.17 de | 4.30 ± 0.18 b | 1.38 ± 0.08 bc | 5.69 ± 0.26 bc | 0.89 ± 0.04 de |
|  | 500 | 90.06 ± 4.39 e | 956.66 ± 39.95 de | 6.95 ± 0.30 ef | 4.04 ± 0.16 bc | 1.23 ± 0.03 cd | 5.27 ± 0.19 cd | 0.86 ± 0.04 de |
| KS | 0 | 217.98 ± 15.04 a | 2033.84 ± 167.80 a | 15.11± 1.45 ab | 5.51 ± 0.20 a | 1.58 ± 0.08 a | 7.09 ± 0.13 a | 1.06 ± 0.03 ab |
|  | 500 | 145.08 ± 4.56 c | 1536.88 ± 19.42 b | 12.96 ± 0.08 bc | 3.65 ± 0.09 c | 1.17 ± 0.05 d | 4.77 ± 0.12 d | 0.81 ± 0.01 e |
| QT | 0 | 168.26 ± 4.95 b | 1948.14 ± 119.83 a | 18.39 ± 2.75 a | 4.34 ± 0.07 b | 1.31 ± 0.04 cd | 5.43 ± 0.07 bc | 0.99 ± 0.01 bc |
|  | 500 | 115.45 ± 0.74 d | 939.43 ± 24.61 e | 6.98 ± 0.24 ef | 4.14 ± 0.03 b | 1.26 ± 0.02 cd | 5.39 ± 0.06 bc | 0.88 ± 0.01 de |
| *P*-value | Cd | ^****^ | ^****^ | ^****^ | ^****^ | ^****^ | ^****^ | ^****^ |
|  | S | ^****^ | ^****^ | ^****^ | ^***^ | ns | ^***^ | ^****^ |
|  | Cd×S | ns | ^***^ | ^**^ | ^***^ | ns | ^***^ | ^*^ |

**Note:** Data indicate means ± SE (n = 3). Different letters behind the values in the same column indicate significant difference between the treatments. *P*-values of the ANOVA of Cd treatment (Cd), soil (S) and their interaction (Cd × S) are indicated. ^*^*P* < 0.05. ^**^*P* < 0.01. ^***^*P* < 0.001. ^****^*P* < 0.0001. ns, not significant. ML, Maliangou village in Liaoyang city. DS, Desheng village in Panjin city. XS, Xishan village in Chaoyang city. KS, Kaoshantun village in Xinmin city. QT, Qianertaizi village in Xinmin city.

**Table S5** Principal component analysis (PCA) of physiological response in the roots, stems, and leaves of apple plants grown in the soils sampled from five apple orchards subjected to 0 or 500 μM CdCl_2_ treatments for 70 days.

| Variables | PC1 | PC2 | PC3 | PC4 | PC5 | PC6 | PC7 |
| --- | --- | --- | --- | --- | --- | --- | --- |
| Root dry mass | -0.19 | 0.16 | 0.10 | -0.14 | 0.08 | 0.07 | -0.01 |
| Stem dry mass | -0.20 | -0.04 | 0.08 | -0.20 | -0.08 | -0.06 | 0.07 |
| Leaf dry mass | -0.15 | -0.08 | 0.15 | -0.15 | -0.01 | -0.30 | 0.15 |
| Stem length | -0.16 | 0.02 | -0.24 | -0.17 | -0.10 | -0.04 | 0.07 |
| Total root length | -0.17 | 0.19 | 0.09 | -0.12 | 0.08 | 0.11 | -0.07 |
| Total root surface area | -0.18 | 0.16 | 0.12 | -0.12 | 0.02 | 0.09 | -0.09 |
| Total root volume | -0.17 | 0.08 | 0.17 | -0.11 | -0.07 | 0.01 | -0.13 |
| Chl a | -0.18 | -0.08 | 0.11 | 0.17 | 0.11 | 0.20 | 0.03 |
| Chl b | -0.19 | -0.04 | 0.06 | 0.15 | 0.09 | 0.16 | -0.01 |
| Chl (a+b) | -0.19 | -0.08 | 0.10 | 0.16 | 0.10 | 0.19 | 0.03 |
| Carotenoid | -0.18 | -0.05 | 0.16 | 0.13 | 0.14 | 0.09 | 0.04 |
| Root Cd concentration | 0.22 | 0.13 | 0.07 | 0.08 | -0.04 | 0.06 | -0.09 |
| Stem Cd concentration | 0.22 | -0.01 | 0.11 | 0.05 | -0.04 | 0.12 | -0.01 |
| Leaf Cd concentration | 0.21 | -0.02 | 0.09 | 0.13 | 0.00 | 0.12 | 0.16 |
| Total Cd content | 0.19 | 0.17 | 0.09 | 0.09 | -0.04 | -0.03 | -0.14 |
| Root O_2_^•−^ | 0.14 | 0.06 | -0.08 | -0.24 | 0.11 | -0.09 | 0.20 |
| Stem O_2_^•−^ | 0.18 | -0.05 | 0.04 | -0.03 | 0.19 | -0.04 | 0.14 |
| Leaf O O_2_^•−^ | 0.17 | 0.08 | 0.08 | -0.22 | 0.19 | -0.07 | -0.04 |
| Root H_2_O_2_ | 0.16 | 0.09 | 0.22 | 0.01 | 0.02 | -0.14 | -0.20 |
| Stem H_2_O_2_ | 0.17 | 0.09 | 0.13 | 0.07 | 0.12 | 0.17 | 0.08 |
| Leaf H_2_O_2_ | 0.10 | 0.25 | 0.13 | -0.02 | 0.18 | -0.03 | -0.12 |
| Root MDA | 0.15 | 0.07 | -0.22 | -0.05 | -0.19 | 0.06 | 0.06 |
| Stem MDA | 0.16 | 0.03 | -0.06 | 0.09 | -0.17 | 0.20 | 0.03 |
| Leaf MDA | 0.10 | 0.20 | 0.18 | 0.11 | 0.11 | 0.06 | 0.12 |
| Root proline | 0.21 | -0.11 | 0.01 | 0.02 | -0.04 | -0.01 | 0.03 |
| Stem proline | -0.06 | -0.19 | 0.11 | 0.25 | 0.01 | -0.07 | -0.13 |
| Leaf proline | 0.02 | -0.16 | -0.05 | 0.11 | 0.04 | 0.28 | -0.16 |
| Root phenolics | -0.11 | 0.04 | -0.21 | 0.26 | 0.07 | -0.09 | -0.01 |
| Stem phenolics | 0.01 | 0.02 | -0.35 | 0.06 | 0.00 | 0.08 | -0.13 |
| Leaf phenolics | -0.11 | 0.20 | -0.14 | 0.17 | -0.04 | -0.11 | 0.14 |
| Root ASC | -0.03 | -0.08 | -0.23 | 0.28 | -0.03 | -0.15 | 0.02 |
| Stem ASC | 0.08 | -0.01 | 0.08 | 0.11 | -0.13 | -0.23 | -0.36 |
| Leaf ASC | 0.06 | 0.18 | -0.05 | -0.14 | 0.04 | 0.27 | 0.18 |
| Root T-SH | 0.04 | 0.22 | -0.07 | 0.01 | -0.24 | -0.18 | 0.22 |
| Stem T-SH | 0.03 | 0.17 | -0.31 | 0.10 | 0.14 | -0.02 | -0.09 |
| Leaf T-SH | -0.06 | 0.21 | -0.04 | -0.06 | -0.33 | 0.02 | 0.08 |
| Root SOD | 0.16 | -0.04 | 0.00 | 0.15 | -0.02 | 0.16 | 0.18 |
| Stem SOD | 0.09 | 0.26 | 0.09 | 0.05 | 0.19 | -0.18 | -0.05 |
| Leaf SOD | 0.09 | 0.24 | 0.13 | 0.07 | -0.10 | 0.08 | -0.30 |
| Root CAT | 0.19 | -0.11 | 0.06 | -0.13 | -0.11 | 0.16 | -0.01 |
| Stem CAT | 0.03 | 0.07 | -0.15 | -0.28 | 0.20 | 0.22 | 0.02 |
| Leaf CAT | -0.07 | 0.00 | 0.24 | 0.02 | -0.09 | 0.04 | 0.41 |
| Root APX | 0.17 | -0.19 | -0.07 | -0.08 | 0.11 | -0.01 | 0.05 |
| Stem APX | 0.16 | -0.17 | 0.06 | -0.01 | 0.12 | -0.04 | 0.17 |
| Leaf APX | 0.11 | 0.05 | -0.02 | 0.07 | -0.21 | 0.15 | 0.06 |
| Root POD | 0.11 | -0.25 | 0.01 | -0.11 | 0.13 | -0.23 | 0.02 |
| Stem POD | 0.01 | 0.17 | 0.04 | 0.19 | 0.29 | -0.21 | 0.19 |
| Leaf POD | 0.08 | 0.09 | 0.13 | 0.12 | -0.29 | -0.24 | 0.15 |
| Root GR | 0.10 | -0.29 | 0.07 | -0.03 | 0.04 | -0.09 | -0.06 |
| Stem GR | -0.04 | 0.15 | -0.07 | 0.28 | 0.20 | -0.03 | 0.22 |
| Leaf GR | -0.06 | -0.08 | 0.27 | 0.09 | -0.29 | 0.08 | 0.09 |
| Proportion of variance | 31.36% | 15.19% | 10.52% | 9.69% | 8.01% | 4.87% | 4.18% |

**Figure S1** Superoxide dismutase (SOD) (A1-A3), peroxidase (POD) (B1-B3), catalase (CAT) (C1-C3), ascorbate peroxidase (APX) (D1-D3) and glutathione reductase (GR) (E1-E3) activity in the roots (A1-E1), stems (A2-E2) and leaves (A3-E3) of ‘Hanfu’ apple plants grown in the soils sampled from five apple orchards subjected to 0 (CK) or 500 μM CdCl_2_ (Cd) for 70 d. Data indicate means ± SE (n = 3). Different letters on the bars indicate significant difference between the treatments. *P*-values indicated for ANOVA of Cd treatment (Cd), soil (S) and their interaction (Cd × S) are indicated. ^*^*P* < 0.05. ^**^*P* < 0.01. ^***^*P* < 0.001. ^****^*P* < 0.0001. ns, not significant. ML, Maliangou village in Liaoyang city. DS, Desheng village in Panjin city. XS, Xishan village in Chaoyang city. KS, Kaoshantun village in Xinmin city. QT, Qianertaizi village in Xinmin city.


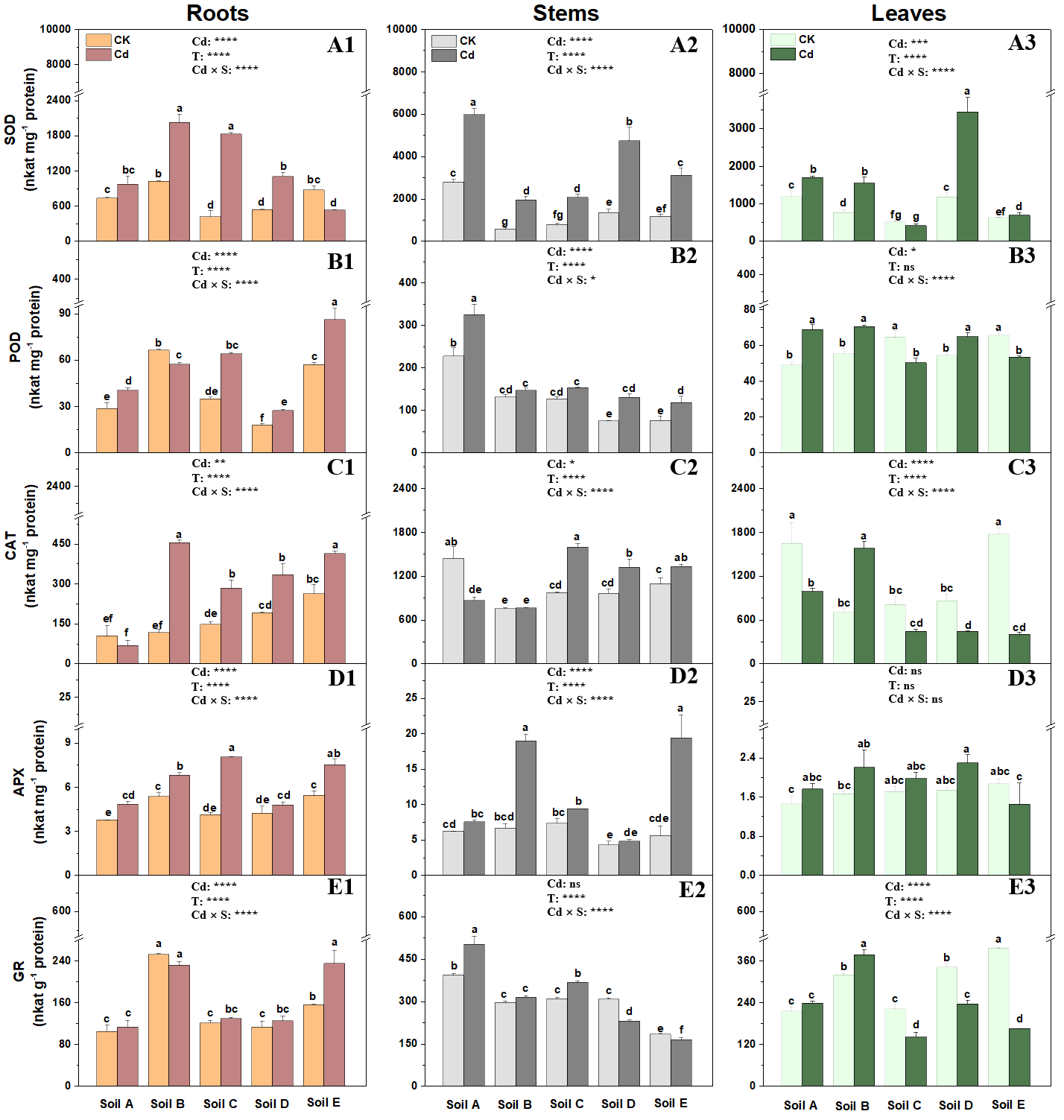

Supplement: Supplementary file 1 [file DataSheet_1.docx]
